# Stem Cell and Regenerative Therapies for the Treatment of Osteoporotic Vertebral Compression Fractures

**DOI:** 10.3390/ijms25094979

**Published:** 2024-05-02

**Authors:** Songzi Zhang, Yunhwan Lee, Yanting Liu, Yerin Yu, Inbo Han

**Affiliations:** 1 Department of Neurosurgery, CHA Bundang Medical Center, CHA University, Seongnam-si 13496, Republic of Korea; szzhang95@gmail.com (S.Z.); poolowen@hotmail.com (Y.L.); dhdtm33@naver.com (Y.Y.); 2 Department of Medicine, School of Medicine, CHA University, Seongnam-si 13496, Republic of Korea; knuvet1029@gmail.com

**Keywords:** osteoporosis, osteoporotic vertebral compression fracture, stem cell, bone regeneration

## Abstract

Osteoporotic vertebral compression fractures (OVCFs) significantly increase morbidity and mortality, presenting a formidable challenge in healthcare. Traditional interventions such as vertebroplasty and kyphoplasty, despite their widespread use, are limited in addressing the secondary effects of vertebral fractures in adjacent areas and do not facilitate bone regeneration. This review paper explores the emerging domain of regenerative therapies, spotlighting stem cell therapy’s transformative potential in OVCF treatment. It thoroughly describes the therapeutic possibilities and mechanisms of action of mesenchymal stem cells against OVCFs, relying on recent clinical trials and preclinical studies for efficacy assessment. Our findings reveal that stem cell therapy, particularly in combination with scaffolding materials, holds substantial promise for bone regeneration, spinal stability improvement, and pain mitigation. This integration of stem cell-based methods with conventional treatments may herald a new era in OVCF management, potentially improving patient outcomes. This review advocates for accelerated research and collaborative efforts to translate laboratory breakthroughs into clinical practice, emphasizing the revolutionary impact of regenerative therapies on OVCF management. In summary, this paper positions stem cell therapy at the forefront of innovation for OVCF treatment, stressing the importance of ongoing research and cross-disciplinary collaboration to unlock its full clinical potential.

## 1. Introduction

**Figure 1:**
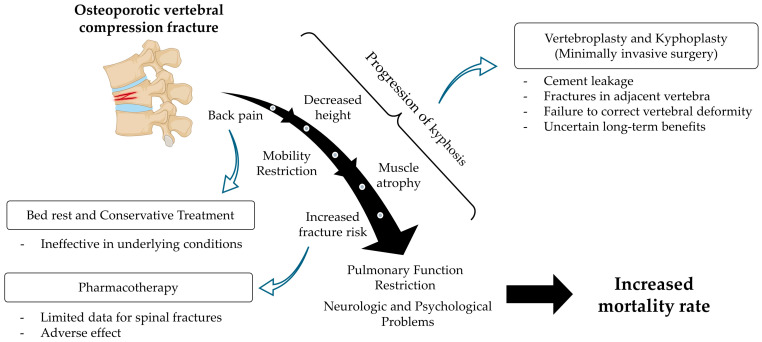
Clinical manifestations, progression, and unmet needs in current treatment approaches for OVCFs.

Osteoporosis is a systemic skeletal disorder characterized by reduced bone density and quality, leading to an increased risk of fractures, especially in the vertebral column [1]. Osteoporotic vertebral compression fractures (OVCFs) are among the most frequent complications of osteoporosis and represent a significant portion of all osteoporotic fractures [2,3]. While the exact percentage varies based on the population studied and the criteria for defining fractures, vertebral fractures are universally acknowledged as a major component [4]. Recent epidemiological studies have shown that OVCFs affect approximately 20% to 30% of individuals over the age of 50 worldwide, and these rates are expected to climb as the population ages [5]. In South Korea, the mean age of occurrence for OVCFs is reported to be 75.15 years, with women experiencing these fractures approximately 3.5 times more frequently than men [6]. Additionally, the mortality rate associated with these fractures is around 24%, with men having a 1.8-fold higher risk of mortality compared to women [7]. These fractures, representing elderly disease, pose a significant health concern, as they substantially affect patient morbidity, mortality, and quality of life (Figure 1) [8]. The high prevalence of vertebral fractures among those with osteoporosis underscores the need for effective prevention, diagnosis, and treatment strategies to manage the condition and improve patient outcomes.

**Figure 2:**
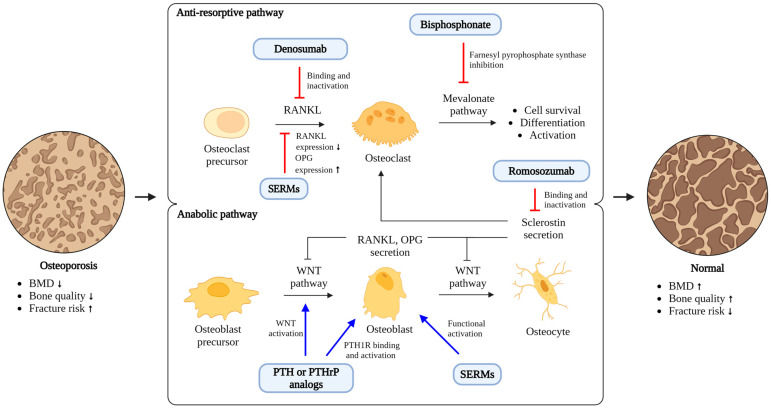
Mechanisms of anti-osteoporotic agents for reducing the risk of osteoporotic vertebral compression fractures. Upward arrows indicate an increase, and downward arrows signify a decrease. BMD, bone mineral density; OPG, osteoprotegerin; PTH, parathyroid hormone; PTHrP, parathyroid hormone-related protein; RANKL, receptor activator of nuclear factor kappa-Β ligand; SERM, selective estrogen receptor modulator.

The current standard of care for OVCFs primarily focuses on managing pain, preventing further bone loss through pharmacotherapy, and, in some cases, surgical intervention to stabilize the fracture [9,10,11]. Pharmacological interventions using anti-osteoporotic agents aim to both prevent the occurrence of fractures and aid in the healing process of osteoporotic fractures. Anti-osteoporotic agents are primarily classified into anti-resorptive agents and anabolic agents, each functioning through distinct mechanisms [12,13,14] (Figure 2). From a preventative standpoint [14], bisphosphonates have been shown to reduce the risks of vertebral, non-vertebral, and hip fractures. Specifically, ibandronate significantly lowers the risk of vertebral fractures. However, its effectiveness in preventing non-vertebral and hip fractures was mainly observed in a post hoc analysis of a subgroup of female patients with a femoral neck T-score below −3. Raloxifene is effective in preventing vertebral fractures only. Teriparatide and abaloparatide have been successful in preventing fractures, with the exception of hip fractures. Denosumab, a RANKL inhibitor, has demonstrated preventative effects across all types of fractures. Romosozumab, a sclerostin inhibitor, is effective in preventing vertebral fractures, but there is less evidence regarding its impact on other types of fractures. Notably, the ARCH study [15] showed that 12 months of treatment with romosozumab followed by 12 months of alendronate significantly reduced the risk of non-vertebral and hip fractures compared to 24 months of alendronate treatment alone. While most anti-osteoporotic medications are effective in preventing vertebral fractures, their effects on the healing process of existing fractures are not uniform. A review of osteoporosis medications, including bisphosphonates, denosumab, selective estrogen receptor modulators (SERMs), and teriparatide, revealed no significant impact on the healing of wrist and hip fractures, with limited data on spine fractures [16]. Denosumab did not hinder the healing of non-vertebral fractures, but comprehensive clinical studies on vertebral fracture healing are lacking. Currently, there is no research available on the effects of SERMs on fracture healing in humans. Teriparatide may slightly accelerate healing times for wrist fractures and has been associated with better pain and functional outcomes in hip fractures, but it did not significantly affect vertebral fracture stability. However, some studies suggest that patients treated with teriparatide for spine fractures experience less pain and significant improvements in vertebral body collapse and kyphotic angle. Clinical studies investigating the effects of romosozumab on vertebral fracture healing have not yet been conducted (Table 1).

Percutaneous vertebroplasty (PVP) and percutaneous kyphoplasty (PKP) are minimally invasive procedures for treating OVCFs, aimed at alleviating pain and stabilizing fractures by injecting bone cement into the vertebral body [17,18]. However, these treatments have several limitations. Firstly, they do not address the underlying osteoporosis, which leaves and increases the risk of subsequent fractures [19,20]. Secondly, the risk of cement leakage, potentially leading to severe complications such as adjacent vertebral fractures, nerve damage, and even pulmonary embolism, underscores the procedure’s inherent risks [21]. Moreover, while both PVP and PKP can provide immediate pain relief, they may not restore vertebral height or correct spinal deformity effectively in all cases [22]. Additionally, while some studies suggest that the outcomes of PVP and PKP may not significantly differ from non-surgical management in the long term, the debate over their long-term efficacy, particularly in terms of pain relief and physical function remains unresolved, highlighting the complexity of managing OVCFs [23,24,25,26]. These limitations of the current management for OVCFs highlight the need for advancements in treatment options, including regenerative therapies, that not only aim to relieve symptoms but also address the broader aspects of osteoporosis and spinal health.

In this comprehensive review, we explore the therapeutic potential of stem cell applications for patients suffering from OVCFs, as well as a detailed examination of the associated challenges and limitations inherent in the current state of stem cell research and its clinical applications. We conducted a search using the keywords “osteoporosis”, “osteoporotic vertebral compression fractures”, “stem cell”, and “bone regeneration” in PubMed (https://pubmed.ncbi.nlm.nih.gov/, accessed on 28 February 2024).

## 2. Regenerative Therapy for Managing OVCFs

The use of stem cell and regenerative therapies in treating OVCFs represents a growing field aimed at overcoming the shortcomings of conventional treatments by promoting bone regeneration and repair. Stem cells, with their distinctive regenerative properties, are leading this innovative approach [27]. The key mechanisms by which stem cells are believed to aid in bone healing involve their differentiation into osteoblasts, the secretion of angiogenic and growth factors that aid in bone remodeling, and the modulation of the local microenvironment to enhance bone regeneration [28,29].

Research in this area predominantly involves mesenchymal stem cells (MSCs), which can be derived from various sources, including bone marrow, adipose tissue, and umbilical cord blood [30,31]. MSCs are favored for their ability to differentiate into bone-forming cells and for their immunomodulatory effects, which can be crucial in treating osteoporotic conditions.

Preclinical studies have shown that MSCs can increase vertebral bone mass and improve the microarchitecture of osteoporotic bone, indicating a promising path for therapeutic intervention. Although still in the preliminary stages, a clinical trial exploring the use of stem cells for OVCFs has yielded promising results. Notably, a phase I/IIa study assessing the safety and efficacy of Wharton’s jelly-derived MSCs (WJ-MSCs) in combination with teriparatide has shown not only feasibility and tolerability but also significant improvements in pain relief, functional recovery, and bone structure in patients with OVCFs. This suggests a potentially valuable direction for regenerative therapy in the treatment of such fractures. Additionally, the development of biomaterial scaffolds as vehicles to deliver stem cells and growth factors directly to the fracture site is another innovative strategy. These scaffolds promote localized bone growth and healing (Table 2).

Despite these advances, several challenges remain. Identifying the optimal source of stem cells, determining their most effective delivery method and dosage, and establishing the long-term safety and efficacy of these treatments are areas that require further investigation [32,33]. Additionally, the variability in patient responses to stem cell therapies underscores the need for personalized treatment strategies that may incorporate genetic, metabolic, and environmental factors influencing bone healing. Although preliminary data from animal models and an early clinical trial are promising, comprehensive clinical studies are essential to confirm the safety, efficacy, and practicality of stem cell-based therapies. This innovative approach has the potential to revolutionize the treatment of OVCFs, offering new hope for patients seeking relief from this debilitating condition.

**Table 2:** Preclinical and clinical studies on stem cell therapy for OVCFs.

Study Aim	Cell Type and Origin	Model	Delivery Method	Therapeutic Outcomes	Ref.
Clinical Study					
- 12-month, open-label, randomized controlled phase I/IIa clinical trial - To evaluate the safety and effectiveness of WJ-MSCs combined with teriparatide for treating patients with OVCFs - Enrolled 20 subjects, randomized into two groups: 10 in the experimental group and 10 in the control group	WJ-MSCs (allogenic)	Human	Intramedullary injection (4.0 × 10^7^ cells) into fracture site, followed by intravenous injection (2.0 × 10^8^ cells) after 1 week	- Significant improvements in the visual analog scale, Oswestry Disability Index, and 36-Item Short Form Survey. - Improved microarchitecture of spine and hip.	[34]
Preclinical Study					
- To develop a biocompatible treatment to address the limitations of vertebroplasty in OVCFs with PMMA-spheroid gel	BM-MSCs (allogenic)	Rat	Direct PMMA-doped MSC spheroid gel implantation into vertebral compression fracture site (1.0 × 10^6^ cells)	- Increase in bone volume and BMD. - Decrease in pain markers in dorsal root ganglia.	[35]
- To evaluate the therapeutic potential of BM-MSCs for managing neural defects associated with VCFs	BM-MSCs (allogenic)	Canine	Percutaneous intraspinal injection (1.0 × 10^6^ cells) every 15 days	- Improvement in loco-motor status and sensory functions in all cases.	[36]
- To investigate bone regeneration in a vertebral defect by MSCs overexpressing BMP-6	Genetically modified BM-MSCs overexpressing BMP-6 (allogenic)	Minipig	Implanted into the vertebral defects (4.0 × 10^6^ cells)	- Increased bone regeneration in response to the implantation of MSCs over-expressing BMP6 compared to minor bone formation in the control. - Enhanced bone regeneration in the BMP6-MSC group versus control group ex vivo.	[37]
- To study the capability of gene-modified adult stem cells overexpressing rhBMP-6 to regenerate vertebral bone in a rat model	Porcine ASCs (xenogeneic)	Rat	Implanted into the vertebral defects (1.0 × 10^6^ cells)	- Considerable defect repair by 2 weeks post implantation, with bone formation rate and final bone volume.	[38]
- To analyze the effects of Sr-β-TCP combined with BM-MSCs or ASCs for spinal fusion - 15 OVX and 15 sham-operated rats divided into groups receiving Sr-β-TCP alone, Sr-β-TCP + BM-MSCs, and Sr-β-TCP + ASCs	BM-MSCs ASCs (syngeneic)	Rat	Direct Sr-β TCP scaffold with MSC implantation at the site of spinal fusion (1.5 × 10^6^ cells)	- Formation of more solid fusion tissue in the Sr-β-TCP + BM-MSC group compared to Sr-β-TCP + ADSCs for both sham and OVX animals. - BMSCs’ superiority over ADSCs in promoting spinal fusion in radiographical scores and histological analysis.	[39]
- To assess the therapeutic potential of the systemic transplantation of MSCs in an age-related osteoporosis model	BM-MSCs (allogenic)	Rat	Intravenous injection (2.0 × 10^6^ to 4.0 × 10^6^ cells)	- Improved bone quality and microarchitectural competence. - Long-term engraftment and increased bone formation.	[40]
- To demonstrate the effectiveness of autologous BM-MSCs combined with porous β-TCP in repairing bone defects in the medial femoral condyle of osteoporotic goats	BM-MSCs (autologous)	Goat	Direct MSCs combined with porous β-TCP implantation into medial femoral condyle defects	- Improved bone formation and critical-sized bone defect repair in osteoporotic conditions. - Significant integration of MSC-β-TCP complex with the surrounding bone.	[41]
- To investigate the effect of an intra-bone marrow injection of BM-MSCs on femur bone mass in osteoporotic female rats	BM-MSCs (autologous)	Rat	Intra-bone marrow injection of BM-MSCs into the femurs of osteoporotic rats (7.5 × 10^5^ cells)	- Increased femur bone mass in treated rats compared to untreated osteoporotic rats. - Similar trabecular bone percentage in treated rats to that of healthy control rats.	[42]
- To determine the effects of BM-MSCs on BMD and mechanical strength in the femurs of ovariectomized rats	BM-MSCs (allogenic)	Rat	Direct injection (1.0 × 10^7^ cells) into each femur	- Significantly increased BMD in BM-MSC-injected femurs versus controls. - Increased mechanical strength with sustained improvements in rats receiving a second injection at 24 weeks.	[43]
- To evaluate whether the introduction of MSCs into sites at risk of osteoporosis in rabbits subjected to OVX can improve the architecture and mechanical properties of bone	BM-MSCs (autologous)	Rabbit	MSCs embedded in calcium alginate gels transplantation into the cancellous space of the distal femur (5.0 × 10^6^ cells)	- Increased BMD in treated femurs. - Increased trabecular thickness and improved microstructures, including newly formed osteoid. - Stronger biomechanical stiffness.	[44]
- To address joint replacement complications due to osteoporosis with a three-dimensional inorganic–organic supramolecular bioactive interface combining a three-dimensional printed porous metal scaffold and a multifunctional supramolecular polysaccharide hydrogel encapsulating BM-MSCs and BMP-2	BM-MSCs (autologous)	Rabbit	Implanted the bioactive interface containing encapsulated BMSCs and/or BMP-2 within the supramolecular hydrogel-filled pTi scaffold into distal femur defects (2.0 × 10^5^ cells)	- Induced proliferation and osteogenic differentiation of BM-MSCs. - Promoted integration of the metal microspore–bone interface both in vitro and in vivo.	[45]
- To investigate the effect of BM-MSCs modified with bFGF on bone regeneration in distraction osteogenesis in rabbits.	BM-MSCs with and without bFGF gene modification (autologous)	Rabbit	Injection of BM-MSCs-(1.0 × 10^7^ cells) with or without bFGF modification into the distraction gaps of the mandibles of rabbits	- Improved bone formation and mineralization with the highest BMD and bone mineral content observed in the bFGF-modified BM-MSC group.	[46]
- To evaluate the efficacy of OPG gene-modified BM-MSCs combined with an HA scaffold in treating critical-sized mandibular defects in osteoporotic rats induced by OVX	BM-MSCs modified to express OPG (autologous)	Rat	OPG gene-modified BM-MSC seeding on HA scaffold implantation (2.0 × 10^5^ cells/cm^2^) into mandibular defects	- Improved bone formation and mineralization in the defect area. - Increased BMD and mineralized volume and reduced osteoclastogenesis.	[47]
- To investigate the feasibility of using CCB coated with BM-MSCs-sheet as a three-dimensional scaffold material in bone repair tissue engineering	BM-MSCs (allogenic)	Rat	- Implantation of CBB coated with allograft BMSC sheets (over 10^7^ cells) in a sandwich structure for cranial defects	- Enhanced osteogenic differentiation and mineralized formation of the CBB-BMSC-sheet combination both in vitro and in vivo. - Significantly higher mRNA expressions of osteogenic markers such as BMP-2, b-FGF, Col1a1, OSX, and Runx-2.	[48]
- To evaluate the effectiveness of SrHA scaffolds, engineered with ASC, on osteogenesis and osteointegration in an osteoporotic sheep model	ASCs (allogenic)	Sheep	Implantation of ASCs on SrHA scaffolds in distal femur defects	- Increased osteogenic activity and mature lamellar bone formation. - Higher regeneration ratio and bone volume and improved osteointegration.	[49]
- To assess the ability of autologous ASCs to improve bone regeneration in a rabbit model of osteoporosis by promoting osteogenesis and reducing adipogenesis	ASCs (autologous)	Rabbit	Implantation of ASCs encapsulated in calcium alginate gel (5.0 × 10^6^ cells) into the distal femurs	- Increased BMD and new bone formation. - Improvements in bone volume/total volume, connectivity density, and trabecular number metrics	[50]

OVCF, osteoporotic vertebral compression fracture; Ref., reference; WJ-MSCs, Wharton’s jelly-derived mesenchymal stem cell; BM-MSCs, bone marrow-derived mesenchymal stem cell; rhBMP-6, recombinant human bone morphogenetic protein-6; ASCs, adipose-derived stem cell; PMMA, polymethyl methacrylate; OVX, ovariectomized; Sr, strontium; TCP, tricalcium phosphate; pTi, porous titanium alloy; bFGF, basic fibroblast growth factor; OPG, osteoprotegerin; HA, hydroxyapatite; CCB, calcined bovine bone; Col1a1, collagen Type 1 alpha 1 chain; OSX, osterix; SrHA, strontium hydroxyapatite.

## 3. Therapeutic Mechanism of MSCs in OVCF Treatment

MSCs have emerged as a promising therapeutic option for osteoporosis and OVCFs, with conditions characterized by reduced bone strength and an increased risk of fractures. The application of MSCs in this context is based on their unique biological properties, which include multipotency, the ability to self-renew, and immunomodulatory effects [51]. This literature review aims to aggregate contemporary research on the therapeutic applications of MSCs, with an emphasis on elucidating their mechanisms of action in the treatment of osteoporosis and OVCFs, even though the accurate mechanisms are not yet fully understood. Research has shown that MSCs promote bone formation by directly differentiating into osteoblasts and secreting growth factors such as bone morphogenetic protein (BMP), vascular endothelial growth factor (VEGF), and transforming growth factor-beta (TGF-β). These factors not only promote the osteogenic differentiation of MSCs but also stimulate the proliferation of resident osteoprogenitor cells [32,52,53,54].

In the context of osteoporosis, the therapeutic efficacy of MSCs extends beyond mere bone regeneration. MSCs have been demonstrated to modulate the bone marrow microenvironment, affecting the equilibrium between bone formation and resorption. This modulation is facilitated through the regulation of signaling pathways, including the Wnt/β-catenin pathway, which is pivotal in osteoblast differentiation and function [55]. Furthermore, MSCs possess immunomodulatory properties that may alter the inflammatory environment within the bone marrow. This alteration has the potential to counteract the inflammation-associated acceleration of bone loss commonly observed in osteoporosis [56,57]. Parallel to research on osteoporosis, studies on OVCFs have underscored the potential of MSC-based therapies, which promote bone healing, alleviate pain, and improve functional outcomes.

In addressing the challenges presented by OVCFs, the reduced bone quality at the core of these conditions calls for a treatment strategy that goes beyond mere symptomatic relief. Such a strategy requires an intervention that addresses the underlying issue of bone fragility. MSCs, with their inherent ability to form bone, are at the forefront of promising therapeutic approaches in this area. The importance of MSCs is further highlighted by the intricate interplay of specific protein alterations, genetic losses, and the inflammatory environment, which together contribute to the development of osteoporosis by affecting MSC function. Significant insights into the role of MSCs in osteoporosis have been gained from various studies. Liu et al. pointed out the age-related decrease in autophagy, particularly targeting fatty acid binding protein 3 (FABP3), which favors fat cell formation over bone formation within MSCs. Consequently, the buildup of FABP3 hinders the ability of MSCs to differentiate into bone-forming cells, leading to osteoporosis [58]. This finding clarifies a key molecular pathway where therapeutic intervention could improve MSC bone formation and slow the progression of osteoporosis. In relation to genetics, the research by Deng et al. has revealed the effects of KDM4B gene deletion, which significantly impairs the self-renewal of MSCs and hastens their depletion [59]. This genetic change contributes to the acceleration of skeletal aging and the worsening of osteoporosis, identifying a genetic factor that could be targeted to potentially restore MSC health and function. Moreover, Cai et al. investigated the dynamic interactions between MSCs and their cellular environment under osteoporotic conditions, finding that increased mitochondrial transfer from macrophages to MSCs leads to higher expressions of proinflammatory genes [60]. This not only reduces the bone-forming abilities of MSCs but also emphasizes the pivotal role of the inflammatory milieu in the pathology of osteoporosis. By modulating these interactions, there is potential to enhance MSC-driven bone formation and alleviate osteoporotic alterations in the vertebrae [32,61].

Understanding the complex molecular mechanisms that govern the differentiation of MSCs into either adipocytes or osteoblasts is crucial, especially since the propensity of MSCs to differentiate into adipocytes rather than osteoblasts is associated with the development of osteoporosis. This differentiation process is tightly controlled by key transcription factors, including Runx2 [62], osterix [63], PPARγ [64], and C/EBPα [65]. These transcription factors play a critical role in determining the fate of MSCs, steering them toward becoming either osteoblasts or adipocytes by altering gene expression patterns within the cells [66]. Recent studies have highlighted the significant role of microRNAs (miRNAs) in the differentiation of MSCs. These small, non-coding RNA molecules are crucial in deciding a cell’s fate by targeting transcription factors essential for osteogenic and adipogenic differentiation. Often, miRNAs promote adipogenic differentiation at the expense of osteogenic differentiation by suppressing the expression of osteogenesis-related transcription factors. This shift in balance toward adipogenesis contributes to the pathophysiology of osteoporosis within the intricate network of signaling pathways and regulatory molecules [67]. Moreover, extensive research has emphasized the important roles of BMPs [68,69] and the WNT signaling pathway [70,71] in the bidirectional differentiation of MSCs. These pathways exhibit complex and variable effects on MSC differentiation into osteoblasts and adipocytes, influenced by factors such as dosage and the specific context of differentiation. BMPs, for instance, are known to encourage osteoblast differentiation and bone formation. In contrast, the role of WNT signaling is more complex, capable of supporting both osteogenesis and adipogenesis depending on the signaling context and interactions with other molecular cues [62,72].

While the exact mechanisms by which MSCs aid in the treatment of OVCFs are not yet fully understood, it is recognized that they play a crucial role in the bone repair process. This includes involvement in the reactive phase, the reparative phase, and the remodeling phase of osteoporosis. Furthermore, the anti-inflammatory and immunomodulatory properties of MSCs are critically important in the healing process, particularly in managing inflammation, which is a key aspect of OVCF pathology [73]. By modulating immune responses, MSCs may foster an environment that is more favorable for bone healing and regeneration, thus highlighting their therapeutic potential in the treatment of osteoporosis and OVCFs. This insight not only deepens our understanding of MSC biology but also paves the way for the development of targeted therapies that leverage the regenerative powers of MSCs to treat osteoporosis and its associated complications.

## 4. The Impact of MSCs on the Healing of OVCFs

Normal bone tissue has a remarkable capacity for self-repair and regeneration restoring its structural and physiological functions after a fracture [74]. This complex biological process of fracture healing is coordinated by a range of cellular participants, including osteocytes, osteoblasts, osteoclasts, and MSCs, as well as numerous microenvironmental factors such as cytokines, chemokines, growth factors, and the complex intracellular and extracellular pathways that facilitate bone induction [75,76]. MSCs, with their multidirectional differentiation potential, homing capabilities, and paracrine effects, are pivotal in this regenerative process. Endogenous MSCs can migrate to the fracture site and differentiate into osteogenic cells, thereby enhancing bone formation and repair [32,33]. However, the degree to which exogenously administered MSCs directly differentiate and contribute to bone regeneration is still debated. In vitro studies have consistently demonstrated the strong proliferation, viability, and osteogenic differentiation potential of exogenous MSCs. In contrast, in vivo studies have shown improvements in bone regeneration parameters, but tracking studies have indicated that only a very small percentage of systemically injected MSCs successfully engraft at the fracture site [37,77]. The hostile microenvironment, largely due to the inflammatory response to injury, poses a significant challenge to cell survival [77,78]. Some studies have suggested that MSCs may not directly differentiate into bone cells but may instead temporarily become chondrocytes [79,80]. These chondrocytes produce an extracellular matrix, which forms the cartilage template for subsequent bone formation. After rapid proliferation, the matrix mineralizes, leading to chondrocyte apoptosis as nutrients become scarce. Blood vessels then invade these spaces, bringing stem cells that differentiate into osteoblasts and osteocytes, crucial for bone deposition. Beyond these direct or indirect differentiation pathways, MSCs also exert therapeutic effects by secreting a range of microenvironmental factors and bioactive molecules. These include regulatory factors and signaling peptides that modulate cell metabolism, immunity, proliferation, migration, death, and nutrition, all of which contribute to cellular homeostasis and create a more favorable microenvironment for fracture healing [81]. While the exact mechanisms by which MSCs aid in the treatment of OVCFs are not yet fully understood, significant insights have been gained by studying the roles of MSCs throughout the three stages of fracture healing: the reactive phase, the reparative phase, and the remodeling phase [82].

As individuals age, the regenerative capacity of MSCs in the bone marrow notably declines. Aging MSCs exhibit decreased proliferation rates and a reduced ability to differentiate into osteoblasts, which are essential for bone formation. Instead, there is an increased tendency for these cells to differentiate into adipocytes, a shift that contributes significantly to the pathophysiology of osteoporosis and increases the risk of fractures. Therapeutic interventions targeting MSCs aim to restore or enhance their regenerative capabilities [83,84]. One approach is the administration of exogenous MSCs, which are often derived from younger, healthier donors and can potentially compensate for the aging-related decline in endogenous MSC populations. These exogenous MSCs can be engineered to express higher levels of regenerative cytokines or to possess greater osteogenic potential, thereby promoting bone repair and regeneration more effectively. Another strategy involves the use of pharmacological agents that stimulate the endogenous MSCs to overcome their senescent state and enhance their regenerative functions. For example, treatments with drugs that activate the Wnt signaling pathway can promote osteogenic differentiation and inhibit the adipogenic differentiation of MSCs [85]. Additionally, anti-senescent therapies that clear senescent cells or modulate inflammatory pathways can rejuvenate the aged MSC population, improving their functionality and therapeutic efficacy. It is crucial to consider that while treatments may rejuvenate or supplement MSC functions, the complexity of the aging bone marrow microenvironment often requires a combination of approaches to achieve significant therapeutic outcomes. Furthermore, understanding the interactions between MSCs and other cells in the bone marrow, such as immune cells and endothelial cells, is essential for developing comprehensive treatment strategies that address not only the symptoms but also the underlying causes of bone degeneration associated with aging.

The initial stage in the healing process of OVCFs, known as the reactive phase, is predominantly characterized by the onset of inflammation and the formation of hematoma. This phase is crucial for setting the stage for subsequent healing and regeneration. MSCs play a vital role during this period through the expression of a wide array of chemokine receptors. These receptors are instrumental in facilitating the migration of MSCs to the fracture site, guided by chemokine signals. Concurrently, this phase witnesses a local and systemic surge in pro-inflammatory cytokines, which drives the recruitment of immune cells to the injury site, incites inflammation in the surrounding soft tissues, and kick-starts the differentiation of osteogenic progenitor cells, thereby initiating the process of skeletal regeneration [52,53]. The cytokine milieu produced at the site of injury, including BMP, tumor necrosis factor-alpha (TNF-α), and VEGF, plays a dual role. These cytokines are involved in orchestrating bone resorption through osteoclasts and facilitating bone growth through osteoblasts. Interleukin-17 (IL-17) also contributes to this complex regulatory network by increasing the efficiency of bone formation. Specifically, BMP is known to promote the differentiation of MSCs into osteogenic cells, thereby playing a crucial role in bone repair and regeneration, while VEGF primarily stimulates vascular cells, ensuring adequate blood supply and nutrient delivery to the healing bone [32]. A significant hallmark of the reactive phase is the elevated presence of reactive oxygen species (ROS), predominantly generated by granulocytes infiltrating the fracture hematoma [86]. These ROS play a critical role in activating and recruiting additional inflammatory cells to the site, thus facilitating the initiation of the repair process. However, an excessive accumulation of ROS can potentially hinder the natural progression of bone repair by inducing cellular damage and oxidative stress, making the regulation of ROS levels a critical aspect of the healing process. At the heart of the body’s defense against oxidative stress is nuclear factor erythroid-2 related factor 2 (NRF2), a pivotal regulator that activates the cellular antioxidant response [61]. NRF2 achieves this by binding to antioxidant response elements within the DNA, thereby inducing the expression of a host of essential genes involved in mitigating oxidative stress. Disruption in the NRF2 pathway can lead to exacerbated macromolecular damage within cartilage calluses, impeding the proliferation and differentiation of chondrogenic progenitor cells and ultimately delaying the healing process [87]. Conversely, the overexpression of NRF2 has been shown to increas the stemness qualities of MSCs, promoting their differentiation into osteoblasts and thereby supporting bone regeneration [88].

The reparative phase of bone healing is crucial for recovery from OVCFs and is characterized predominantly by the formation of cartilaginous and bony calluses. This phase begins with an inflammatory response that attracts progenitor cells to the fracture site, leading to the development of a soft callus. The subsequent processes of vascularization, resorption, and ossification contribute to the formation of a hard bone callus, which is essential for restoring bone integrity [61]. The mobilization and recruitment of MSCs from local tissues and the systemic circulation are vital in this phase, participating in a complex molecular interplay that results in the production of collagen matrices, which are essential for tissue scaffolding [54]. Signaling molecules, especially those from the TGF-β superfamily, such as TGF-β2, TGF-β3, and growth differentiation factor-5 (GDF-5), play a crucial role in chondrogenesis and endochondral ossification. Concurrently, bone morphogenetic proteins (BMP-5 and BMP-6) promote cell proliferation, which is critical for intramembranous ossification, underscoring the multifaceted roles of these factors in the dynamics of bone healing. As the reparative process advances, cytokines such as macrophage colony-stimulating factor (M-CSF), a receptor activator of nuclear factor kappa B ligand (RANKL), and osteoprotegerin (OPG), along with TNF-α, become increasingly important. These molecules coordinate the recruitment and activity of bone cells and osteoclasts, ensuring the resorption of mineralized cartilage and the formation of new bone tissue. This complex regulatory network facilitates the transition from soft to hard callus, ultimately replacing the calcified cartilage with lamellar bone and restoring mechanical strength and integrity to the affected vertebra [70]. Increasing the osteogenic potential of MSCs through microenvironmental signals such as TNF-α, coupled with the critical role of macrophage activation in MSC recruitment and differentiation, highlights the intricate interplay between immune and regenerative processes in bone healing. The proposal by Pajarinen et al. to target the interaction between MSCs and macrophages offers a novel therapeutic approach to improving fracture healing outcomes, demonstrating the ongoing development of strategies that leverage the regenerative capabilities of MSCs for treating OVCFs [89].

Bone remodeling is the final stage of the fracture healing process, which can take several months to a few years to complete, depending on the fracture’s severity and type. This phase is crucial for restoring the biomechanical stability of the bone, signifying the transition from a temporary matrix to mature bone tissue. During remodeling, a second phase of bone resorption occurs, where the initially formed woven bone, resulting from endochondral ossification, is systematically replaced by the more organized and mechanically superior lamellar bone. Simultaneously, the shape of the intramedullary canal is carefully reconstructed, indicating a return to the bone’s normal anatomical and functional state [32]. For successful bone remodeling, two essential conditions must be satisfied: an adequate blood supply to the region and a progressive increase in mechanical stability to support the stresses and strains of everyday activities. The importance of proper vascularization is paramount, as it not only provides necessary nutrients and oxygen but also aids in the removal of waste products, thus fostering an ideal environment for the regeneration of bone tissue.

**Figure 3:**
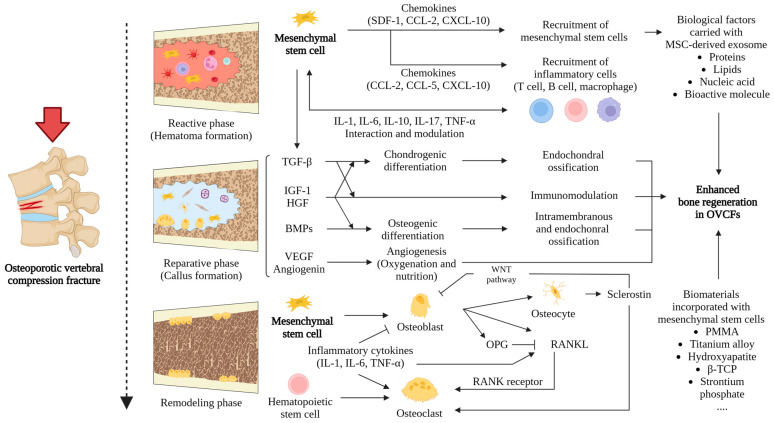
Mechanisms of action in stem cell-based therapy for osteoporotic vertebral compression fractures (OVCFs).

Throughout the various stages of fracture healing in OVCFs, the function of MSCs adapts, although the boundaries between each phase are not clearly defined. Initially, during the reactive phase, MSCs exhibit primarily anti-inflammatory properties by releasing cytokines such as IL-1, IL-6, IL-10, and TNF-α [90,91]. They also attract immune cells, including T cells, B cells, and macrophages, to modulate the immune response. In the reparative phase that follows, MSCs transition to regenerative roles, secreting a range of bioactive molecules that facilitate bone tissue repair and regeneration. This includes growth factors like insulin-like growth factor-1, TGF-β, and VEGF, which are crucial for cell proliferation and vascularization [91,92]. Additionally, MSCs produce angiogenin to promote new blood vessel formation and hepatocyte growth factor to support the regeneration of various tissues. During the remodeling phase, MSCs demonstrate the capacity to differentiate into multiple cell types relevant to bone and other tissues, such as chondrocytes, osteocytes, adipocytes, fibroblasts, smooth muscle cells, and cells of other tissue-specific types (Figure 3).

## 5. MSC-Based Therapies for Treating OVCFs

Research on bone marrow (BM)-derived MSC therapy for OVCFs has utilized both experimental animal models and human surgical interventions to evaluate its efficacy. The success and credibility of this therapy hinge on factors such as the source of BM-MSCs, the methods of cell transplantation, and the inherent variability in animal models and human OVCF patients [53,93,94]. Preclinical studies involving the transplantation of BM-MSCs into ovariectomized (OVX) animal models, including rats [42], mice [95], rabbits [44], and goats [41], have shown improvements in bone strength. These models mimic the bone weakening caused by estrogen deficiency, thereby offering insights into potential treatments for OVCFs resulting from hormonal imbalances [96]. The interventions have been promising in increasing bone robustness in these models, indicating potential strategies for combating osteoporosis related to hormonal fluctuations [97].

Research conducted by Uejima et al. [43] and Wang et al. [44] demonstrated that the injection of BM-MSCs into the distal femurs of animals helped preserve the mechanical properties of the bones, as evidenced through biomechanical testing. Yu et al. [95] found that BM-MSC transplantation decreased TNF-α levels and increased T-cell apoptosis, BMD, the trabecular number, and the bone volume fraction. These results suggest that BM-MSCs play a role in immunoregulation and could be effective in treating osteoporosis caused by estrogen deficiency. Furthermore, studies by Kiernan et al. [40] and Ichioka et al. [98] documented long-term engraftment and significant bone formation in models of age-related osteoporosis following MSC transplantation. Taken together, these findings support the use of BM-MSC transplantation as a promising treatment option for both estrogen-deficient and age-related osteoporosis [96]. Consequently, BM-MSC-based therapy is emerging as a potential intervention for the prevention and management of OVCFs, aiming to alleviate pain, reduce symptoms, and promote recovery [99].

Several studies on BM-MSCs focusing on vertebral defects warrant attention. Pelled’s research introduced a novel, potentially minimally invasive method for bone regeneration in OVCFs using allogeneic gene-modified MSCs. This study involved MSCs engineered to express bone morphogenetic protein 6 (MSC-BMP6), which were encapsulated in a fibrin gel and implanted into vertebral defects in a large animal pig model, resulting in increased bone formation within the defects [37]. Sharun et al. concentrated on evaluating the therapeutic potential of allogeneic BM-MSCs (aBM-MSCs) for addressing neural deficits associated with OVCFs in dogs with vertebral compression fractures [36]. Their research suggests that the intraspinal delivery of aBM-MSCs, in conjunction with supportive therapy, could effectively manage neural deficits in canines with non-displaced OVCFs. These studies underscore the versatility of BM-MSCs in various medical applications and the ongoing efforts to improve MSC-based therapies. However, these studies have limitations, including small sample sizes and the lack of an osteoporosis model, such as the ovariectomized model, which could influence the outcomes of vertebral fracture recovery. Additionally, while the use of fibrin gel as a carrier can promote the osteogenic differentiation of MSCs under certain conditions, it presents challenges due to its inadequate biomechanical properties, particularly its mechanical strength, which is vital for weight-bearing structures like the spine [100]. Therefore, further research is needed, employing adequate sample sizes and investigating alternative biomaterials or carriers to increase the efficacy and safety of stem cell therapies.

The first clinical trial of MSCs for OVCFs combined teriparatide treatment with injections of Wharton’s jelly-derived MSCs (WJ-MSCs), administered both intramedullary and intravenously. At the 12-month follow-up, this approach led to significant improvements in pain, function, and quality of life, confirming the clinical advantage of WJ-MSCs in improving the bone structure of OVCF patients [34]. Although osteoporosis is a systemic disorder, the systemic transplantation of MSCs in humans has demonstrated limited spontaneous engraftment at the lesion sites, rendering MSC monotherapy less effective [78]. This restricted engraftment ability raises questions about the therapeutic effectiveness of systemically transplanted MSCs. The efficacy of stem cell therapy is now believed to be primarily due to the paracrine secretion of bioactive molecules that attract cells to the lesion site and promote tissue repair through angiogenesis, immunomodulation, and the activation of resident stem cells [101]. Therefore, the targeted delivery of MSCs to the fracture site is crucial for the reconstruction of bone architecture in OVCF patients. For systemic MSC therapies, it is vital to promote homing and engraftment by employing strategies such as bone-related hormones, cytokine pretreatment, hypoxia-induced chemokine receptor expression, genetic modifications, and mechanical stress. Methods to improve the migration and homing of MSCs include the use of bone-related hormones like parathyroid hormone, cytokine pretreatment with agents such as IL-6 and hepatocyte growth factor, short-term hypoxia exposure to increase chemokine receptors like C-X-C chemokine receptor type 4 (CXCR4) and CX3CR1, genetic modifications to express specific receptors and adhesion molecules, and the application of mechanical stress [77,102,103]. Reflecting on these advancements, a clinical study on OVCFs highlighted the practical challenges and potential risks of stem cell therapies. Notably, a case of pulmonary embolism following intravenous stem cell injection was reported. Such events emphasize the range of risks associated with stem cell therapy, which can vary from minor issues such as fever and pain at the injection or surgical site to major complications like thromboembolism, fibrosis, and oncogenesis [104]. Considering the significant risk of MSCs becoming trapped in the lungs after systemic administration and the serious consequences of pulmonary embolism, it is prudent to perform follow-up chest CT scans for the effective management and monitoring of potential pulmonary complications.

The use of MSCs as a standalone treatment for OVCFs presents several challenges. While a direct intrabone implantation of BM-MSCs has demonstrated cell persistence for up to six months post-implantation in mouse models, a significant number of these cells perish within the first 48 h following transplantation [105,106]. The survival and long-term effectiveness of the transplanted MSCs are hindered by issues such as host immune responses, insufficient migration to the injury site, and the inhospitable conditions present at the injury location [33]. Despite these obstacles, numerous studies have documented significant improvements in bone formation parameters in fracture models after MSC transplantation, with benefits observed over prolonged periods. These positive results are primarily due to the paracrine effects of MSCs, which are exerted through secreted factors and extracellular vesicles. These paracrine mechanisms contribute to creating a favorable microenvironment for healing by promoting immunomodulation, angiogenesis, anti-apoptotic activities, and antioxidative responses, thereby enhancing the functionality of native MSCs. Furthermore, the revitalization of the body’s own MSCs in patients with osteoporosis through either direct interaction or the transfer of information from healthy, externally sourced MSCs is crucial for therapeutic success.

In research on OVCFs, a limited range of MSC types have been predominantly utilized, including WJ-MSCs, BM-MSCs, and adipose-derived MSCs (ASCs), with less exploration into other stem cell types. Studies have varied significantly in terms of MSC dosage, routes of administration, sizes of vertebral defects, experimental models, and methods for assessing therapeutic outcomes. Although it may be premature to determine the absolute therapeutic efficacy of stem cell therapy in this context due to the small sample sizes of some studies, the majority of research indicates positive outcomes. Improvements have been observed in bone turnover markers, BMD, and computed tomography (CT) metrics, such as connectivity density and the bone volume index, in experimental groups treated with MSCs. Therefore, further research into the use of MSCs for managing OVCFs is encouraged, with an emphasis on the need for standardized methodologies to evaluate the therapeutic impact of stem cell therapy on OVCFs.

Transitioning from these promising research findings to the clinical application of stem cell therapy for OVCFs presents a unique set of challenges. For stem cell therapy to be effective in treating OVCFs, it is essential to not only ensure cell proliferation for large-scale production but also to maintain the quality of the MSCs. However, with successive cell culture passages, cellular senescence increases, which raises concerns about reduced therapeutic effectiveness, an increased risk of malignancy, and the potential for ectopic tissue formation. Kiernan et al. [40] demonstrated that low-passage, unmodified MSCs could achieve long-term bone marrow engraftment in a mouse model of age-related osteoporosis. This finding underscores the challenge of balancing the cost and scale-up production for commercialization. To address the challenges of MSC-based therapy, the exploration of exosomes, which are the focus of extensive research and biomaterials that are already in clinical use, seems promising. These strategies may offer viable solutions for overcoming the hurdles associated with stem cell therapy for OVCFs.

Expanding on the theme of innovative approaches to promote the regeneration of endogenous MSCs, it is pivotal to explore advanced strategies that might include gene therapy, parabiosis, and other novel interventions that aim to rescue impaired MSCs or rejuvenate aged MSCs, thus enhancing their functionality in the treatment of OVCFs [107,108]. Gene therapy holds significant promise for enhancing the regenerative capabilities of endogenous MSCs by directly modifying their genetic material. Techniques such as CRISPR/Cas9 can be employed to edit genes involved in aging and senescence. Parabiosis, the process of surgically joining two organisms so they share a circulatory system, has been explored in research as a method to study aging and rejuvenation. Studies have shown that exposing aged mice to the circulatory system of younger mice can reverse signs of aging in various organs and tissues, including the bone marrow. This rejuvenating effect is believed to be due to factors in the younger blood that restore the function of aged MSCs. Incorporating biomaterials that mimic the extracellular matrix (ECM) of bone can also promote the activation and differentiation of endogenous MSCs. These scaffolds can be designed to release bioactive molecules over time, thus providing a supportive environment that mimics young, healthy bone marrow. This approach can encourage MSCs not only to proliferate but also to maintain their osteogenic differentiation potential, effectively contributing to the healing of OVCFs. Altering the local microenvironment or ‘niche’ of MSCs in the bone marrow to simulate a younger, more regenerative state is another innovative approach [109]. Each of these approaches represents a cutting-edge frontier in regenerative medicine and bone health, promising new avenues for the effective treatment of osteoporosis-related complications.

## 6. MSC-Derived Exosomes in the Treatment of OVCFs

Exosomes are extracellular vesicles that possess a complete membrane structure and range in diameter from 30 to 150 nm. They play a crucial role in facilitating the transport of materials and the transmission of information between cells, and they are characterized by their low immunogenicity and their ease of storage and delivery [110]. Stem cells release exosomes through paracrine mechanisms, and these exosomes inherit similar biological properties from their parent cells. However, they are considered to be safer, more stable, and more efficient. This makes them particularly promising for clinical applications where safety and stability are of the utmost importance. Exosomes are particularly adept at transporting and regulating complex signaling molecules. Despite the potential of stem cell therapy, there are ongoing concerns about its safety, including the risks of oncogenicity, thromboembolism, fibrosis, and ethical issues related to the sourcing of cells. Exosomes have emerged as a viable alternative that addresses these concerns, as evidenced by meta-analysis studies [111]. The shift toward exosome-based therapies is indicative of a broader trend in regenerative medicine toward approaches that are safer and more ethically acceptable. In cases where cell transplantation is not feasible, stem cell-derived exosomes offer new possibilities for tissue regeneration and repair, marking them as a significant advancement in the field of stem cell technology [112,113].

Recent studies have highlighted the potential of exosomes derived from mesenchymal stem cells (MSC-Exos) as a promising alternative to direct MSC therapy (Figure 3). MSC-Exos have demonstrated effectiveness in preventing bone loss and promoting bone remodeling processes, such as osteogenesis, osteoclastogenesis, immunomodulation, and angiogenesis, in both in vitro and in vivo studies [114]. They play a pivotal role in musculoskeletal healing, particularly in the regeneration of cartilage and tendons [115]. Additionally, exosomes have been found to accelerate fracture healing in animal models [116] and show promise in treating common joint disorders, including osteoarthritis and osteochondral injuries. Hui et al. [117] showed that exosomes from BM-MSCs increased various osteogenic activities in MG-63 cells, a human osteosarcoma cell line commonly used in osteogenic research, and prevented bone loss in ovariectomized rats. Our research further corroborates that extracellular vesicles, especially exosomes from glycoprotein non-melanoma clone B (GPNMB)-modified BM-MSCs, can reduce bone loss in ovariectomized rats. Consequently, MSC-Exos hold significant promise for preventing OVCFs by halting the progression and deterioration of osteoporosis.

During fracture healing, the reactive and reparative phases are pivotal, with the subsequent remodeling phase being crucial for improving the biomechanical properties of the bone. Optimal healing requires the regulation of osteogenic differentiation and osteoblast proliferation, maintaining a balance between osteoblasts and osteoclasts, and promoting the inhibition of apoptosis, angiogenesis, and immunomodulation [111]. MSCs-Exos transport proteins, lipids, nucleic acids, and various bioactive molecules that can regulate and facilitate the healing process. Notably, miRNAs within exosomes have been identified as critical for fracture repair [118]. For example, miR-148a and miR-218 in osteoblast precursor-derived exosomes suppress the expression of V-musculoaponeurotic fibrosarcoma oncogene homolog B (MAFB), thus regulating the balance between osteoblasts and osteoclasts, while promoting osteoblast differentiation when derived from human BM-MSCs exosomes [119,120]. MSCs-Exos containing miR-29b-3p significantly increase the volume and density of the callus bone, aiding in fracture repair [121]. Additionally, MSCs-Exos with miR-199b, miR-218, miR-135b, miR-221, and miR-148a are known to regulate osteogenesis [122]. Research is advancing on methods such as incubation, electroporation, sonication, and transfection for selectively loading target molecules into exosomes [123]. Furthermore, dysregulated inflammation during the reactive phase can hinder healing by increasing bone resorption and reducing formation. T cells, which are essential for osteoclast and osteoblast formation and immune activity in osteoporosis, are modulated by MSCs-Exos, underscoring their immunomodulatory role. Therefore, MSCs-Exos at fracture sites create a favorable environment for bone healing and regeneration, promoting recovery and ensuring the maintenance of bone quality and strength post-recovery.

Despite the promising potential of exosome therapy for treating OVCFs, direct research on their role in the healing of OVCFs remains limited, with current studies remaining predominantly at the preclinical or early clinical trial stages for other disease [124]. Additionally, challenges in scaling up the production of targeted exosome therapies include extended production times, concerns over purity, variability in exosome characteristics due to differing culture conditions and cell passages, and the complexity of production, all of which contribute to low yields. Among separation methods such as size-exclusion chromatography, ultrafiltration, immunoaffinity, microfluidics, and co-precipitation, ultracentrifugation is seen as the most cost effective for exosome isolation but is hampered by potential contamination risks, time-intensive processes, and low yields [125]. Identifying effective and scalable exosome isolation and purification techniques remains a pivotal challenge for realizing their therapeutic potential. Therefore, there is a critical need for more research to identify additional target molecules beneficial for bone regeneration, develop standardized protocols for the scalable and homogenous production of MSC-derived exosomes loaded with these targets, find methods for their long-term storage, and invigorate the nascent field of exosome therapy research for OVCF treatment.

The safety profile of MSC-derived exosomes, combined with their capacity to therapeutically modulate essential processes such as osteogenesis, angiogenesis, and inflammation, highlights their potential as an effective alternative to MSC-based therapies. This novel strategy shows promise for improving treatment outcomes in patients with osteoporosis and managing OVCFs. Addressing the challenges associated with their production could fully unleash the therapeutic capabilities of MSC-derived exosomes. Their ability to transport bioactive molecules and affect crucial cellular functions makes them a significant asset in regenerative medicine, particularly for bone-related disorders.

## 7. Biomaterials Loaded with Stem Cells in the Treatment of OVCFs

Biomaterials play a pivotal role in facilitating bone tissue repair, offering versatility in form and ranging from three-dimensional structures to fluid-like substances suitable for implantation [126]. They are classified by their chemical composition into polymers, ceramics, metals, and extracellular matrices, with each category exhibiting distinct characteristics [127]. Synthetic polymers, such as poly(lactic acid) (PLA) and polycaprolactone (PCL), as well as natural ones like collagen, fibrin, and chitosan, vary in biodegradability and typically demonstrate low mechanical strength. Ceramics, which include β-tricalcium phosphate, hydroxyapatite, and calcium sulfate, are excellent for bone integration but have limitations in load-bearing applications due to their lower compressive strength and fracture toughness [128]. Currently, poly(methyl methacrylate) (PMMA) is widely used as bone cement in vertebral augmentation procedures, such as vertebroplasty or kyphoplasty, offering rapid improvement in strength and pain relief in areas affected by OVCFs. For clinical success, biomaterials must be biocompatible, osteoinductive, and osteoconductive; support cell adhesion and three-dimensional proliferation; and provide mechanical stability [129]. Recent advancements are centered on developing three-dimensional scaffolds that facilitate new bone formation, often augmented with growth factors or cells to promote osteogenic differentiation [130]. The survival and functionality of transplanted stem cells, which are crucial for harnessing the properties of biomaterials, depend on a supportive microenvironment rich in oxygen and nutrients [131]. However, the altered microenvironment in osteoporosis calls for targeted therapeutic strategies for enhancement. Considering the vital role of vasculature in the tissue microenvironment, promoting angiogenesis is essential for fracture healing [132]. This can be achieved through three-dimensional printed scaffolds with specifically designed channel and pore sizes to guide angiogenesis, and these may also incorporate stem cells or growth factors such as VEGF [133].

In osteoporotic conditions, the diminished functionality of MSCs impedes bone regeneration, complicating the healing process of fractures, particularly when using implanted biomaterials. To address this issue, the strategy of implanting biomaterial scaffolds enriched with exogenous stem cells and bioactive molecules has emerged as a promising solution for managing osteoporotic fractures [30]. This approach was designed to compensate for the reduced activity of MSCs and to provide structural support at the site of the fracture. While research on the integration of stem cells and bioactive molecules into scaffolds for osteoporotic applications is still in its infancy, early studies have reported encouraging results. These findings indicate the potential for biomaterials to effectively treat osteoporotic fractures, with positive outcomes observed across a variety of scaffold materials, animal models, and fracture locations.

The process of osteointegration in OVCFs is multifaceted, involving not only stem cells and osteoprogenitor cells but also bone-forming growth factors [134]. The concurrent transplantation of stem cells and growth factors has been shown to promote bone integration. For instance, the use of porous titanium alloy scaffolds in conjunction with rabbit BM-MSCs and BMP-2 has been shown to promote bone ingrowth and osteointegration, positively modifying the osteoporotic microenvironment [45]. Additionally, BM-MSCs are recognized for their potential as therapeutic cells, including their application in gene therapy to deliver genes that can modify the disease environment [46]. A novel approach involves BM-MSCs engineered with the OPG gene, used in combination with hydroxyapatite scaffolds, to modulate the activity of bone-forming cells and bone-resorbing osteoclasts. This strategy shows promise for the reconstruction of bone defects in osteoporosis [47].

Integrating BM-MSCs with various biomaterials has yielded promising results for bone regeneration, especially in osteoporotic conditions (Figure 3). The combination of mixed β-tricalcium phosphate (β-TCP) and strontium phosphate (Sr_3_(PO_4_)_2_)-loaded BM-MSCs for posterolateral spinal fusion has led to enhanced bone formation and increased fusion efficacy in both osteoporotic and non-osteoporotic models [39,135]. In a similar vein, implanting calcined bovine bone with BM-MSCs into the calvarial defects of OVX rats significantly improved new bone formation compared to using MSCs alone [48]. Adipose-derived mesenchymal stem cells (AD-MSCs) have also shown promise in bone healing and osteogenesis. For example, a strontium hydroxyapatite scaffold combined with sheep AD-MSCs improved osteogenesis and osteointegration, aiding the healing process in osteoporotic bone [49]. Furthermore, employing a calcium alginate gel as a carrier for AD-MSCs in the distal femur of ovariectomized rats not only promoted BM-MSC proliferation and osteogenic differentiation in vitro but also increased bone regeneration in vivo [50]. These studies underscore the significant potential of merging biomaterial scaffolds with exogenous stem cells to accelerate ossification, encourage bone ingrowth, and facilitate osteointegration, particularly under the challenging conditions of osteoporosis. This strategy not only presents a promising path for enhancing bone regeneration but also highlights the critical importance of innovative material science in the advancement of stem cell therapies for the treatment of osteoporotic bone injuries.

In a notable study by Ko et al. [35], MSCs were integrated with PMMA, a material commonly used in vertebroplasty or kyphoplasty for OVCFs. The study employed glycol chitosan and oxidized hyaluronate as carriers for the MSCs, using a rat femur injury model to emulate the predominantly trabecular bone structure of the human spine. The results showed that the combination of PMMA with MSCs promoted osteogenesis and angiogenesis, improved osteoconduction with the host bone, and decreased pain markers, such as transient receptor potential vanilloid-1 (TRPV-1) and ionized calcium-binding adapter molecule-1 (Iba-1). These findings underscore the promising potential of integrating stem cell therapy with PMMA, highlighting the necessity for further research to investigate long-term outcomes and potential side effects. The success of the study suggests the clinical viability of stem cell-based biomaterials for managing OVCFs, representing a significant advancement in addressing the limitations of current PMMA-based treatments in vertebroplasty and kyphoplasty. This innovative approach merges developments in biomaterial science with stem cell therapy, pointing to a promising direction for the development of more effective and sustainable treatments for fractures related to osteoporosis.

## 8. Summary

MSC-based therapy offers significant promise for treating OVCFs by promoting bone regeneration. This is achieved through various mechanisms such as osteogenic differentiation, the secretion of cytokines and chemokines, the recruitment of cells, angiogenesis, immunomodulation, and antioxidative activities. Collectively, these processes expedite fracture healing and improve the recovery milieu at the site of the fracture [56].

Nonetheless, the translation of MSC therapy into clinical practice is fraught with multiple challenges. A primary obstacle is the need for high-quality, large-scale MSC production, as clinical applications require between 10^10^ and 10^12^ cells per batch. Traditional two-dimensional culture methods are inadequate for meeting these demands, which underscores the potential of three-dimensional culture systems and microcarriers as more efficient alternatives [136]. Furthermore, the heterogeneity of MSCs—affected by tissue origin, genetic background, and donor age—complicates standardization and can influence the effectiveness of treatments, introducing variability in clinical outcomes [103,137]. MSCs derived from induced pluripotent stem cells present a potential solution by promoting homogeneity and standardization [138,139]. Additional challenges include the costs associated with MSC therapy, difficulties with engraftment and cell viability, and concerns about long-term effects [38], as well as potential adverse events such as thromboembolism, fibrosis, oncogenesis, and ethical issues. To address these challenges, innovative strategies are required, including the direct injection of MSCs into lesion sites, the use of exosome-based therapies, and the development of novel biomaterials.

While advancements in MSC-based therapies have shown promise for treating bone defects, research focusing on vertebral defects, particularly within osteoporosis models, is still limited. Given the substantial burden that OVCFs place on patients, it is essential to direct future studies toward evaluating MSC therapies that are specifically designed for OVCFs. Targeted research is imperative to provide solid evidence of both efficacy and safety, which will ultimately lead to the development of optimal treatment strategies. This approach will not only expand the therapeutic options for OVCFs, but also deepen our understanding of how to manage this significant health concern, thereby improving outcomes for patients with osteoporosis-related vertebral injuries.

## Figures and Tables

**Table 1:** Comparison of the efficacy of anti-osteoporotic agents in reducing fracture risk and treating OVCFs [14,16].

Anti-Osteoporotic Agents	Anti-Fracture Efficacy			Impacts on OVCF Healing
	Vertebral	Non-Vertebral	Hip	
Anti-resorptive agents				
- Alendronate	+	+	+	No significant clinical outcomes
- Risedronate	+	+	+	
- Ibandronate	+	+ **	NE *	
- Zoledronate	+	+	+	
- Raloxifene (SERM)	+	NE *	NE *	No clinical studies
- Denosumab	+	+	+	Limited clinical studies
Anabolic agents				
- Teriparatide	+	+	NE *	Improvement of vertebral body collapse and kyphotic angle ****
- Romosozumab	+	+ ***	+ ***	No clinical studies

* No sufficient evidence. ** Effective in female patients with femoral neck bone mineral density T score < −3.0. *** 12 months of romosozumab followed by 12 months of alendronate. **** No significant improvement in stability parameter. OVCF, osteoporotic vertebral compression fracture; SERM, selective estrogen receptor modulator.

## Abbreviations

OVCFs	osteoporotic vertebral compression fractures
MSCs	mesenchymal stem cells
PVP	percutaneous vertebroplasty
PKP	percutaneous kyphoplasty
SERMs	selective estrogen receptor modulators
BMD	bone mineral density
miRNAs	microRNAs
BMP	bone morphogenetic protein
TNF-α	tumor necrosis factor alpha
VEGF	vascular endothelial growth factor
IL-17	interleukin 17
ROS	reactive oxygen species
NRF2	nuclear factor erythroid-2 related factor 2
M-CSF	macrophage colony-stimulating factor
RANKL	receptor activator of nuclear factor kappa B ligand
OPG	osteoprotegerin
TGF-β	transforming growth factor-beta
IL-1	interleukin-1
BM-MSCs	bone marrow-derived mesenchymal stem cells
OVX	ovariectomized
aBM-MSCs	allogenic bone marrow-derived mesenchymal stem cells
WJ-MSCs	Wharton’s jelly-derived MSCs
MSCs-Exos	exosomes sourced from MSCs
GPNMB	glycoprotein non-melanoma clone B
BMSCs	bone marrow stem cells
Β-TCP	β-tricalcium phosphate
OPG	osteoprotegerin
AD-MSC	adipose tissue-derived mesenchymal stem cell
PMMA	polymethyl methacrylate
